# Consumers’ participation in information-related activities on social media

**DOI:** 10.1371/journal.pone.0250248

**Published:** 2021-04-23

**Authors:** Su-Jung Nam, Hyesun Hwang

**Affiliations:** 1 Department of Home Economics Education, College of Education, Jeonju University, Jeollabuk-do, Republic of Korea; 2 Department of Consumer Science, Convergence Program for Social Innovation, College of Social Sciences, Sungkyunkwan University, Jeollabuk-do, Republic of Korea; West Pomeranian University of Technology, POLAND

## Abstract

This study examined the relationship between consumers’ characteristics and social media use, focusing on consumers’ information-related activities such as creating content, sharing information, and providing feedback on information. The results showed that consumers’ creating content, sharing information, and providing of feedback on information were affected by their digital literacy, need for cognition, and self-esteem. Information literacy and need for cognition had positive effects on engagement in these information-related activities, but self-esteem exerted a negative effect.

## Introduction

Academic interests have given weight to the negative and problematic use of the Internet and social media [1–4]. Internet addiction or the heavy usage of information communication technologies (ICTs) has been associated with negative emotions and closely linked to low self-esteem [4–8]. However, since consumers are considered important participants in the formation of a better information ecosystem, it is necessary to consider how they contribute to the processes of creating content, sharing information, and providing feedback on information.

Social media are interactive computer-mediated technologies that facilitate the creating content or sharing of information, ideas, career interests, and other forms of expression via virtual communities and networks [9–10]. Consumers usually access social media services via web-based applications (apps) on desktops and laptops, or by downloading apps that offer social media functionality on their mobile devices (e.g., smartphones and tablets). As consumers engage with these electronic services, they create highly interactive platforms through which individuals, communities, and organizations can share, co-create, discuss, participate in, and modify user-generated or self-curated content posted online. Social media form a collective intelligence by allowing people to participate in the process of generating and sharing information based on a system centered around the real-time flow of information [11–13]. Activities for the purposes of creating new information, sharing information with others, and giving feedback in the information production process can be viewed as positively contributing to collective intelligence and fostering a sense of community engagement, even if there is no direct reward for participants [12,13]. These activities may reinforce social media’s role as an information source based on widely shared ideas. Individual consumers can contribute to the flow of information on social media by sharing existing information with others; providing feedback, such as expressing their opinions, to generate information collaboratively; and creating new information on their own.

Therefore, in this study, consumers’ information-related behaviors on social media are classified into three types: creating content, sharing information, and providing feedback on information. In this study, we examined the effects of consumers’ digital literacy, need for cognition, and self-esteem on these three information-related behaviors. The results of this study will extend prior work that addresses the relationship between consumers’ characteristics and social media use from an information production perspective, and enhance our understanding of the role of consumers, who are major contributors to the flow of information on social media and who elucidate their information-related activities with different behavioral levels.

## Theoretical background

### Demographic characteristics and social media

Consumers produce and share information through social media and extend the relationships they form with others. In previous studies of social media users, demographic characteristics were generally the most actively considered variables [14–17]; additionally, psychological variables such as cognitive needs and self-esteem have also begun to be considered [14,18,19]. In a study that reviewed many previous studies related to the characteristics of Facebook users, it was suggested that variables such as demographic characteristics, personality characteristics, and self-esteem were strongly associated with Facebook use [14]. In addition, the needs of cognition are also regarded as an important variable in information related behavior [18,19].

Some studies have focused on various demographic characteristics, but most studies also include other variables in empirical models [20]. For example, one study examined the influence of self-esteem along with demographic characteristics such as gender, age, and education on the addictive use of social media [21].

Common demographic variables included in studies related to social media use include age, gender, income level, and education level. According to the results of these studies, lower age, women, and higher income and education levels are associated with a higher likelihood of using social media [14,18,20,21].

### Digital literacy

The rapid spread of the Internet and smartphones has led to a greater variety of usage gaps, which have been discussed only in terms of accessibility in the digital environment [22,23]. Digital literacy is related to the ability to locate, organize, understand, evaluate, and analyze information using digital technology. It involves a working knowledge of current advanced technology and an understanding of how such technology can be used. Furthermore, digital literacy involves a consciousness of the technological forces that affect culture and human behavior [24]. Digitally literate people can communicate and work more efficiently, especially with those who possess the same knowledge and skills. The study of digital literacy is concerned with wider aspects beyond computer literacy that are associated with learning how to effectively find, use, summarize, evaluate, create, and communicate information while using digital technologies [25,26].

It is expected that this digital literacy will affect social media use because a digitally literate person will possess a range of digital skills; knowledge of the basic principles of computing devices; skills in using computer networks; the ability to engage in online communities and social networks while adhering to behavioral protocols; the ability to find, capture, and evaluate information; an understanding of the societal issues raised by digital technologies such as big data; and critical thinking skills [27]. As a result, digital literacy can be expected to influence creating content, sharing information, and providing feedback. Therefore, Hypotheses 1a to 1c were posited based on previous studies:

**Hypothesis 1a.** Digital literacy has a positive effect on creating content.
**Hypothesis 1b.** Digital literacy has a positive effect on sharing information.
**Hypothesis 1c.** Digital literacy has a positive effect on providing feedback on information.

### Need for cognition

Consumers’ information-related activities on social media can be associated with their motivation to process new information or issues. This can be viewed from the perspective of the need for cognition, which refers to an individual’s tendency to engage in and enjoy activities that require thinking [28], and which is related to an individual’s tendency to pursue a cognitive stimulus and to the degree of involvement in information or cognitive effort [29]. Some individuals have relatively little motivation for cognitively complex tasks. These individuals are described as having a low level of need for cognition. Other individuals consistently engage in and enjoy cognitively challenging activities and are referred to as having a high level of need for cognition. Consumers with a high level of need for cognition are more motivated to process many messages than those with a low level of need for cognition; hence, the former are more active online [30]. Therefore, the need for cognition has been treated as an important variable that leads to computer-mediated communication [18,31,32]. People with a high level of need for cognition tend to prefer media with high information delivery [33]. Furthermore, the need for cognition has a positive effect on the degree of web use, which is a useful variable in predicting the degree of social media use [34]. Consequently, the need for cognition can be expected to influence creating content, sharing information, and providing feedback. Therefore, the following hypotheses were posited based on previous studies.

**Hypothesis 2a.** The need for cognition has a positive effect on creating content.
**Hypothesis 2b.** The need for cognition has a positive effect on sharing information.
**Hypothesis 2c.** The need for cognition has a positive effect on providing feedback on information.

### Self-esteem

Self-esteem is an individual’s subjective evaluation of their own worth, encompassing one’s beliefs about oneself, as well as emotional states such as triumph, despair, pride, and shame [35]. Self-esteem encompasses positive or negative self-evaluations [36]. In research on Internet addiction, self-esteem has been discussed as a factor affecting the degree of Internet use and Internet addiction [14]. For teenagers with low self-esteem, in particular, online spaces can be attractive because they enable users to conceal their true identities and project their ideal selves instead [37]. Since online activities can reduce the stress and discomfort of face-to-face situations, people with low self-esteem tend to enjoy the Internet [38]. Since a computer-mediated communication environment can lower the barriers to communication with others, people with low self-esteem are highly likely to use such an environment as a channel of communication [39]. As a result, self-esteem can be expected to influence creating content, sharing information, and providing feedback. Therefore, the following Hypotheses 3a to 3c were posited based on previous studies.

**Hypothesis 3a.** Self-esteem has a negative effect on creating content.
**Hypothesis 3b.** Self-esteem has a negative effect on sharing information.
**Hypothesis 3c.** Self-esteem has a negative effect on providing feedback on information.

## Materials and methods

### Data

This study used the Korean Media Panel (KMP) of the Korea Information Society Development Institute (KISDI) for 2015 [40]. The KISDI is a government-affiliated institute with an aim of developing communications infrastructure by collecting, surveying, and researching a variety of data and information about IT policy and business. The KISDI has conducted annual surveys of the KMP that include items regarding media usage status, media consumption patterns, and emotional state. The KMP was approved by the Korean government in 2010, and has since been conducting this panel survey on media use and tracking the responses. Therefore, the survey is designed to maintain the same questionnaire for each survey, with modifications to reflect changes in related industries and media use trends based on previous studies and opinions of the advisory committee. Informed consent was obtained from all respondents for participation in the survey at the data collection phase by the KISDI. This study did not require formal consent since it is a retrospective study with secondary data and the data were analyzed anonymously. KMP is appropriate for the purposes of this study because it contains various items regarding media usage status, media consumption patterns, and emotional state, among others. Specifically, the KMP data were collected from a stratified sample of 9,555 consumers aged 19 years and older and living in 16 metropolitan city regions in Korea. In this study, 5,842 adults over 19 years of age (m = 4.54, SD = 11.13, range [min = 19, max = 59]) who used social media at least once during a given month were analyzed.

### Measurement

For consumers’ information-related activities on social media, creating content was measured according to participants’ experience of posting new self-created information, sharing information was measured by their experience of posting information received from other people for the purpose of sharing it with others, and providing feedback on information was measured by participants’ experience of recommending or rating other people’s posts. The items were developed by the KISDI and have been applied to the KMP since 2010, which is approved by the Korean government as national statistics. These variables are binary; they are treated as 1 if they were experienced during a given month and as 0 if they were not experienced during that month.

For digital literacy, respondents answered 13 yes-no questions about activities related to digital literacy (i.e., viewing or sending a message, playing or downloading a video, clicking a favorite site, visiting an Internet site using the URL bar, conducting an information search, Internet banking, making an online reservation, viewing or sending an email, attaching a file to an email, and downloading an attached file from an email) developed by KISDI to identify respondents’ ability of media usage. Respondents received 1 point for answering yes and 0 points for no.

Need for cognition was assessed using the Korean version of Gim’s self-report questionnaire, Need for Cognition (K-NfC-S) [41], with Cronbach’s α = .90. Fifteen items (“I want to learn more about things I have little knowledge about,” “I enjoy learning about new solutions to problems,” etc.) were scored on a 4-point Likert scale, ranging from 1 = Not concerned at all to 4 = Very concerned. The scores were summed to yield a total score, with a higher score corresponding to a greater need for cognition.

Self-esteem was assessed using Rosenberg’s self-esteem scale [42], which also takes the form of a self-report questionnaire, with Cronbach’s α = .76. Ten items (“I am satisfied with myself,” “I feel that I have a number of good qualities,” etc.) were scored on a 4-point Likert scale, ranging from 1 = Strongly disagree to 4 = Strongly agree. The scores were summed to yield a total score, with a higher score corresponding to greater self-esteem.

### Analysis

To reveal the factors influencing social media behavior, logistic regression was performed using SPSS 24.0, with female, age, years of education, mean of monthly income, digital literacy, need for cognition, and self-esteem as independent variables and creating content, sharing information, and the providing feedback on information as dependent variables. Logistic regression is a statistical model that in its basic form uses a logistic function to model a binary dependent variable. In regression analysis, logistic regression estimates the parameters of a logistic model (a form of binary regression) [43]. Mathematically, a binary logistic model has a dependent variable with two possible values represented by an indicator variable, where the two values are labeled 0 and 1.

## Results

### Participants’ general characteristics

The study participants’ general characteristics are provided in Table 1.

**Table 1:** Participants’ characteristics (*n* = 5,842).

Characteristics	*n*	%	Mean±*SD*
Gender			
Male	2,700	46.2	
Female	3,142	53.8	
Age			41.54±11.13
≤ 29	1,060	18.1	
30–39	1,150	19.7	
40–49	2,011	34.4	
50–59	1,621	27.7	
Education			13.89±2.53^b^
Elementary school	117	2.0	
Middle school	242	4.1	
High school	2,425	41.5	
College	2,927	50.1	
Higher than college	131	2.2	
Monthly income^a^			164.85±162.52
None	1,937	33.2	
≤ 100	435	7.4	
101–200	1,208	20.7	
201–300	1,073	18.4	
≥ 301	1,189	20.3	
Content creation			
Yes	1,230	21.1	
No	4,612	78.9	
Information sharing			
Yes	1,282	21.9	
No	4,560	78.1	
Feedback provision			
Yes	1,215	20.8	
No	4,627	79.2	
Digital literacy			10.77±.58
Need for cognition			2.42±.38
Self-esteem			2.90±.41

^a^ The unit is South Korean 10,000 won (KRW 10,000 = USD 8.81);

^b^ years of education.

Regarding the respondents, the proportion of males is 46.2%, while 53.8% are female. The proportion of all respondents aged 40–49 years is 34.4%, higher than the other age groups. Additionally, the respondents’ average age is 41.54 (SD = 11.13). Regarding the level of education, 50.1% have a college education, followed by a high school education (41.5%), a middle school education (4.1%), a higher than college education (2.2%), and elementary school (2.0%). Additionally, the respondents’ average years of education is 13.89 (SD = 2.53). Regarding monthly personal income, the largest percentage of respondents (33.2%) are in the no-income group, and the lowest percentage (7.4%) are in the KRW ≤ 100 group. Additionally, the respondents’ average monthly income is KRW 164.85 (SD = 162.52). For information-related activities, 21.1% of respondents have experience creating content, 21.9% have experience sharing information, and 20.8% have experience providing feedback on information.

### Factors influencing information-related activities on social media

Results regarding factors influencing creating content, sharing information, and providing feedback are provided in Table 2.

**Table 2:** Logistic regression of creating, sharing, and feedback.

	Creating content				Sharing information				Providing feedback			
	*B*	*SE*	Exp (*B*)	*p*	*B*	*SE*	Exp (*B*)	*p*	*B*	*SE*	Exp (*B*)	*p*
Female (ref. = male)	.026	.078	1.027	.734	-.029	.077	.971	.705	-.079	.078	.924	.310
Age	-.043	.004	.958	.000	-.039	.004	.962	.000	-.049	.004	.952	.000
Years of education	.093	.019	1.098	.000	.084	.018	1.087	.000	.072	.019	1.074	.000
Mean of monthly income	.000	.000	1.000	.420	.000	.001	1.000	.516	.000	.000	1.000	.558
Digital literacy	.185	.020	1.203	.000	.182	.019	1.200	.000	.172	.020	1.187	.000
Need for cognition	.460	.095	1.584	.000	.405	.093	1.500	.000	.402	.096	1.494	.000
Self-esteem	-.540	.087	.582	.000	-.496	.085	.690	.000	-.624	.088	.536	.000
Intercept	-2.750				-2.645				-1.640			
-2Log likelihood	5,271.662				5,459.014				5,233.246			

The results for the influence on creating content indicate that age (B = −.043, SE = .004, Exp (B) = .958, p < .001), years of education (B = .093, SE = .019, Exp (B) = 1.098, p < .001), digital literacy (B = .185, SE = .020, Exp (B) = 1.203, p < .001), need for cognition (B = .460, SE = .095, Exp (B) = 1.584, p < .001), and self-esteem (B = −.540, SE = .087, Exp (B) = .582, p < .001) were statistically significant (−2Log likelihood = 5271.662). Therefore, Hypotheses 1a, 2a, and 3a were supported.

The results for the influence on sharing information show that age (B = −.039, SE = .004, Exp (B) = .962, p < .001), years of education (B = .084, SE = .018, Exp (B) = 1.087, p < .001), digital literacy (B = .182, SE = .019, Exp (B) = 1.200, p < .001), need for cognition (B = .405, SE = .093, Exp (B) = 1.500, p < .001), and self-esteem (B = −496, SE = .085, Exp (B) = .690, p < .001) were statistically significant (−2Log likelihood = 5459.014). Hypotheses 1b, 2b, and 3b were thus supported.

Finally, the results for the influence on the providing feedback on information show that age (B = −.049, SE = .004, Exp (B) = .952, p < .001), years of education (B = .072, SE = .019, Exp (B) = 1.074, p < .001), digital literacy (B = .172, SE = .020, Exp (B) = 1.187, p < .001), need for cognition (B = .402, SE = .096, Exp (B) = 1.494, p < .001), and self-esteem (B = −.624, SE = .088, Exp (B) = .536, p < .001) were statistically significant (−2Log likelihood = 5233.246). Hypotheses 1c, 2c, and 3c were thus supported.

## Discussion

This study advances research on consumers’ social media use by focusing on their behavior as contributing to the information ecosystem on social media. To this end, the influences of digital literacy, the need for cognition, and self-esteem on consumer’s information-related activities (i.e., creating content, sharing information, and providing feedback) were investigated.

The factors that influence creating content, sharing information, and providing feedback were found to be age, years of education, digital literacy, need for cognition, and self-esteem.

First, the lower the age, the lower the creating content (B = -.043, SE = .004, Exp(B) = .958, p < .001), sharing information (B = -.039, SE = .004, Exp(B) = .962, p < .001), and providing feedback (B = -.049, SE = .004, Exp(B) = .952, p < .001). In addition to, the higher the education level, the higher the creating content (B = .093, SE = .019, Exp(B) = 1.098, p < .001), sharing information (B = .084, SE = .018, Exp(B) = 1.087, p < .001), and providing feedback (B = .072, SE = .019, Exp(B) = 1.074, p < .001). These results were consistent with previous studies [14,20,21].

Second, it was found that digital literacy has a positive effect on creating content (B = .185, SE = .020, Exp(B) = 1.203, p < .001), sharing information (B = .182, SE = .019, Exp(B) = 1.200, p < .001), and providing feedback (B = .172, SE = .020, Exp(B) = 1.184, p < .001). In order to effectively operate social media, consumers need to master digital literacy, including the ability to interact with digital information effectively. Such interaction involves familiarity with electronic data, as well as the ability to locate, evaluate, and critically judge the validity, accuracy, and appropriateness of accessed information [44]. With the development of social media, digital literacy is expanding to encompass the concept of media literacy. Some scholars argue that media literacy should include critical analysis, consumption, participation, and information sharing on social media [45,46]. Therefore, in future research, it is necessary to consider the areas of creating content, sharing information, and providing feedback, which were investigated in this study, for inclusion in media literacy.

Third, it was found that need for cognition has a positive effect on creating content (B = .460, SE = .095, Exp(B) = 1.584, p < .001), sharing information (B = .405, SE = .093, Exp(B) = 1.500, p < .001), and providing feedback on information (B = .402, SE = .096, Exp(B) = 1.494, p < .001). A previous study found that people with a high level of need for cognition use social media excessively [47], and a study of senior consumers also found that it has a positive effect on social networking site use [20]. The higher the level of need for cognition, the greater the likelihood of an individual engaging in creating content, sharing information, and providing feedback, which indicates that information is more likely to be produced by people who enjoy the thinking or cognitive effort involved in information processing. This raises the possibility that social media information sources are not regarded as merely peripheral or as simple clues. Although the credibility of social media as an information source can be questioned, it is possible that the information is elaborated upon as more people combine their cognitive efforts [48–50]. Therefore, the value of consumer-driven information on social media will need to be addressed in future research.

Finally, it was found that self-esteem negatively influenced creating content (B = -.540, SE = .087, Exp(B) = .582, p < .001), sharing information (B = −.496, SE = .085, Exp(B) = .690, p < .001), and feedback on information (B = −.624, SE = .088, Exp(B) = .536, p < .001). Previous studies mostly dealt with consumers’ negative behaviors such as Internet addiction or problematic Internet or media use, which are associated with low self-esteem. Thus, there is a solid body of research in this regard [1,5–8]. The results of this study indicate that consumers’ proactive and productive behaviors can also be negatively related to self-esteem. Previous studies offer one possible explanation for this result: The anonymity of the Internet attracts people who are passive or who have difficulty interacting with others [31,45,46]. On social media, users can construct their own profiles allowing them to present their lives and experiences selectively through photographs and texts while exercising a high degree of control over their self-presentation; they can also maintain or develop new relationships with other people [14,47,48]. These features of social media may provide a means for those who negatively evaluate themselves to overcome their low self-esteem. Similarly, prior research has indicated that people with low self-esteem are more likely to adopt social media in a more beneficial way [10,12].

To understand the characteristics of consumers who participate in the production and sharing of information on social media, this study analyzed consumers over a wide age range. In this study, we analyzed young consumers—who are considered the main users of social media—as well as those who are older. Since the different types of social media, which are used differently by age groups, are not separately analyzed, the ability of this study to determine the differences in the types of information production across different social media is limited. Therefore, future research should delineate the types of social media and their different usage characteristics based on different age groups. In addition, this study focused on consumers’ behavior, with the consumers positioned as producers of information only through social media. Therefore, it is necessary to expand the current understanding of social media use as a source of consumer information by dealing with the complex process of information production and use. Furthermore, it is necessary to understand social media’s value as an information source and the background of consumer information processing and decision making in a new media environment.
